# Identification and expression responses of TCP gene family in *Opisthopappus taihangensis* under abiotic stress

**DOI:** 10.3389/fpls.2025.1499244

**Published:** 2025-03-06

**Authors:** Ting Gao, Xiaojuan Zhou, Mian Han, Yuexin Shen, Yimeng Zhang, Qi Wu, Haoyuan Dan, Tingyu Wang, Hang Ye, Li Liu, Min Chai, Yiling Wang

**Affiliations:** ^1^ School of Life Science, Shanxi Normal University, Taiyuan, China; ^2^ Key Laboratory of Resource Biology and Biotechnology in Western China, Ministry of Education, College of Life Sciences, Northwest University, Xi’an, China

**Keywords:** *Opisthopappus taihangensis*, TCP gene family, abiotic stress, gene expression analysis, evolution

## Abstract

The TCP gene family plays pivotal roles in the development and abiotic stress responses of plants; however, no data has been provided for this gene family in *Opisthopappus taihangensis*. Based on *O. taihangensis* genome, 14 *TCP* genes were identified and divided into two classes (I and II). After tandem and segmental duplication/whole-genome duplication (WGD), more loss and less gain events of OtTCPs occurred, which might be related with the underwent purifying selection during the evolution. The conserved motifs and structures of *OtTCP* genes contained light response, growth and development, hormone response, and stress-related cis-acting elements. Different *OtTCP* genes, even duplicated gene pairs, could be expressed in different tissues, which implied that *OtTCP* genes had diverse function. Among OtTCPs, *OtTCP4*, *9* and *11* of CYC clade (Class II) presented a relative wide expression pattern with no or one intron. The three *TCP* genes could be regarded as important candidate factors for *O. taihangensis* in growth, development and stress response. These results provided some clues and references for the further in-depth exploration of *O. taihangensis* resistance mechanisms, as well as those of other unique eco-environment plants.

## 1 Introduction

Transcription factors are one kind of proteins that play essential roles in the growth and development of plants by binding to specific gene promoters or enhancer regions (Katagiri and Chua, 1992). Based on the characteristics of their structural domains, transcription factors may be classified as WRKY (WRKYGQ), SPL (SQUAMOSA promoter-binding protein-like), NAC (NAM, ATAF, and CUC), AP2/ERF (Apetala2/Ethylene Responsive Factor), TCP (TEOSINTE BRANCHED1/CYCLOIDEA/PCF), and various other families (Lehti-Shiu et al., 2017). Among them, TCP transcription factors are specific to plants, cell growth, and cell proliferation (Zhan et al., 2023). Every TCP member has an atypical basic helix–loop–helix (bHLH) secondary structure that made up of two hydrophilic α-helices, a disordered loop, and about 60 amino acid residues (Cubas et al., 1999). This conserved domain is essential for DNA binding, protein interactions, and the regulation of downstream gene expression in the biological processes of plants (Martín-Trillo and Cubas, 2010; Manassero et al., 2013).

As ancient transcription factors that appeared ~650–800 million years ago (Navaud et al., 2007), the genes of TCP family are primarily categorized Class I and II, due to the deletion of four amino acids in the basic domain of Class II (Martín-Trillo and Cubas, 2010). Class I (also referred to as the TCP-P class) contains PCF genes (PCF1 and PCF2), can promote cell proliferation and growth. Class II is TCP-C genes, divided into CYC/TB1 and CIN branches. Different branches may have diverse function. The *TB1* genes play an important role in inhibiting lateral branch growth and male flower formation (Doebley et al., 1997; Dixon et al., 2018), the *CYC* genes are generally involved in the expression of lateral regions of early floral organs and regulates floral symmetry (Luo et al., 1999; Hileman, 2014). Whereas the *CIN* genes are mainly related to leaf morphogenesis (Nath et al., 2003; Palatnik et al., 2003; Crawford et al., 2004; Walcher-Chevillet and Kramer, 2016). At the structure of Class II, some members contain an arginine-rich R domain with an unknown biological function aside from the TCP domain. Most CYC/TB1 members possess a conserved and functionally uncharacterized ECE motif (a sequence of glutamic acid-cysteine-glutamic acid) (Howarth and Donoghue, 2006; Martín-Trillo and Cubas, 2010). From the evolutionary viewpoint, the *CYC/TB1* genes have not been discovered in lycophytes or other early-diverging land plants (Horn et al., 2015). Thus, *CIN* genes might have arisen earlier than *CYC/TB1* genes (Palatnik et al., 2003; Koyama et al., 2007; Horn et al., 2015).

*TCP* genes typically form homodimers or heterodimers with each other to regulate the expressions of target genes (Li et al., 2017). All target genes of *TCPs* contain a highly conserved DNA motif (G(T/C) GGNCCCAC), specifically the core motif (TGGGCC, GCCCR, GG(A/T) CCC) (Kosugi and Ohashi, 2002; Li et al., 2005; Schommer et al., 2008; Aggarwal et al., 2010; Viola et al., 2012; Danisman et al., 2013; Parapunova et al., 2014). Further, TCPs engage with various other transcription factors, such as DELLAs, AS2, ABI4, MYBs, and bHLHs, that can promote flavonoid biosynthesis, trigger effector immunity, respond to abiotic stress, and mediate salicylic acid (SA), jasmonate (JA), auxin, cytokinin (CK), abscisic acid (ABA), and gibberellin (GA) responses (Pruneda-Paz et al., 2009; Li et al., 2012; Steiner et al., 2012; Li and Zachgo, 2013; Tao et al., 2013; Chen et al., 2014; Davière et al., 2014; Marín-De-La-Rosa et al., 2014; Mukhopadhyay and Tyagi, 2015). In *Arabidopsis*, *TCP20* interacts with NIN-like proteins NLP6 and NLP7 to modulate signal transduction pathways, as well as to control root growth (Guan et al., 2017). *AtTCP5*, *AtTCP13*, and *AtTCP17* positively regulate the responses of *Arabidopsis* under high-temperature stress (Han et al., 2019; Zhou et al., 2019). In maize, the natural variation in the *ZmTCP42* promoter is significantly related to drought tolerance. The overexpression of *ZmTCP42* can increase the sensitivity of transgenic *Arabidopsis* to abscisic acid (ABA) and increase its tolerance to drought stress (Ding et al., 2019). In moso bamboo, *PeTCP10* enhances the salt stress tolerance (Xu et al., 2022). Nonetheless, these studies primarily concentrated on the functions and molecular mechanisms of TCPs in model plants (such as *Arabidopsis thaliana*) and agricultural species. Limited researches would be performed on wild and/or non-model plant species.

*Opisthopappus taihangensis* belongs to the family Asteraceae, it is endemic to the Taihang Mountains that span Henan and Shanxi Provinces and typically grows within steep cliff crevices, or on slopes up to ~1000 meters above sea level (Chai et al., 2018; Zhou et al., 2024). Being a cliff species, *O. taihangensis* exhibits good cold and drought resistance and has high ecological and ornamental value, with a large number of flowers and lengthy flowering period (Chen et al., 2022; Han et al., 2024; Zhang et al., 2024c). During drought stress, *O. taihangensis* presents decreased relative water and chlorophyll contents although having a high degree of proline accumulation (Gu et al., 2019). Under longer salt stress exposure times and at higher salt concentrations, *O. taihangensis* survives by engaging redox-regulated antioxidant enzyme mechanisms, including superoxide dismutase (SOD), peroxidase (POD), and catalase (CAT) (Zhou et al., 2024). More, the upregulated genes under salt stress are primarily participated in the processes related to amino acid metabolism, the regulation of transcription factors, ABA signaling pathway, osmolyte metabolism, and antioxidant enzyme activities (Gu et al., 2019; Yang et al., 2020). However, the roles of the transcription factors involved in these responses (e.g., TCPs) are unknown when *O. taihangensis* is under abiotic stress.

For this study, the characteristics of the TCP gene family, including their evolution and diversification in *O. taihangensis* were initially explored using bioinformatics and comparative analyses based on its whole genomics data. Subsequently, the expression levels of *O. taihangensis TCP* genes in distinct tissues under abiotic stress were investigated using RNA-seq data and qRT-PCR. Finally, the potential roles and regulatory pathways of *TCP* genes in response to abiotic stress for *O. taihangensis* were elucidated. The results provided important clues for the further investigation of the endurance mechanisms of *O. taihangensis* in cliff environments, which are foundational for the study of other unique cliff plant species.

## 2 Materials and methods

### 2.1 Identification of *TCP* genes in *O. taihangensis*

With the *O. taihangensis* genome database obtained by our previous study (Ye et al., 2024; Zhou et al., 2024), various strategies were adopted to ensure the integrity (as much as possible) of the TCP gene family in *O. taihangensis*. Firstly, the protein sequences of *TCP* genes in *A. thaliana* were downloaded from TAIR website as queries, the *O. taihangensis* protein sequences were from its genome database. These protein sequences then were used to identify *O. taihangensis TCP* genes by BLAST program with an e value of 1 × 10^-5^, whereas the other parameters were set to default values (NumofThreads: 2, NumofHits: 500, NumofAligns: 250) in TBtools (Chen et al., 2020). After which, the TCP domain was retrieved based on the hidden Markov model (HMM) (PF03634) with Simple HMM Search. Finally, all *TCP* genes were analyzed by the NCBI Batch-CDD tool (Wang et al., 2023b) (https://www.ncbi.nlm.nih.gov/Structure/bwrpsb/bwrpsb.cgi) combining the BLAST and HMM search results, and the genes containing the entire TCP domain were retained.

The identified TCPs were designated as OtTCP + numbers (**Table 1**). Subsequently, the ExPASy (ProtParam) (Duvaud et al., 2021) (http://www.expasy.org/tools/protparam.html) tool was used to evaluate the physicochemical characteristics of the OtTCP proteins, including the number of amino acids (aa), isoelectric point (pI), and molecular weights (MW). And then, the subcellular locations of the OtTCP proteins were predicted using WoLF PSORT (Horton et al., 2007) (https://wolfpsort.hgc.jp/).

**Table 1:** Predicted TCP protein data in *O. taihangensis*.

Gene Name	Amino acids	Mol. Wt (Da)	Isoelectric Point (pI)	Instability Index (II)	Aliphatic Index	Hydropathicity (GRAVY)	Subcellular Localization
OtTCP1	384	41937.36	8.99	47.14	59.51	-0.723	nucleus
OtTCP2	335	38198.39	8.05	51.03	55.01	-0.957	nucleus
OtTCP3	384	43636.82	8.49	70.39	65.55	-0.857	nucleus
OtTCP4	390	43796.8	5.39	49.71	69.03	-0.658	nucleus
OtTCP5	426	45725.23	6.36	56.33	53.47	-0.784	nucleus
OtTCP6	375	41276.05	5.95	43.93	54.29	-0.848	nucleus
OtTCP7	376	40804.91	7	46.02	55.59	-0.675	nucleus
OtTCP8	397	44602.9	6.31	42.28	50.1	-0.906	nucleus
OtTCP9	386	43868.06	6.44	58.74	56.11	-0.998	nucleus
OtTCP10	370	39747.78	6.7	54.81	59.41	-0.638	nucleus
OtTCP11	335	38378.86	9.08	43.54	62.03	-0.855	nucleus
OtTCP12	318	36234.54	9.23	41.56	65.03	-0.803	nucleus
OtTCP13	241	26398.93	6.7	45.55	56.27	-0.776	nucleus
OtTCP14	297	33705.2	9.74	44.16	58.42	-0.884	nucleus

### 2.2 Phylogenetic relationships, gene structures, and conserved motifs of OtTCPs

Using the Clustal X in MEGA (Kumar et al., 2018) with defaulted parameters, multiple sequence alignment (MSA) was conducted by the protein sequences of TCPs in *O. taihangensis*, *A. thaliana* and *Oryza sativa*. The conserved regions of the obtained sequences were subsequently trimmed using trimAl in TBtools (Chen et al., 2020). Then, the rootless phylogenetic tree was constructed using IQ-TREE 2 software (Nguyen et al., 2014) with the maximum likelihood (ML) method and the bootstrap validation parameter 1000. All TCP proteins’ conserved domains and amino acid sequences were compared and examined using the GeneDoc program (Nicholas and Nicholas, 1997).

MEME tool in the MEME SUITE (https://meme-suite.org/meme/tools/meme) online website (Bailey et al., 2009) was employed to examine the motifs (number =10) of the TCP protein sequences of *O. taihangensis*. The relative genetic structural data was obtained from the *O. taihangensis* genome database (GFF file) based on our laboratory. The protein motifs and intron/exon organization were visualized using Gene Structure View (Advanced) in TBtools (Chen et al., 2020).

### 2.3 Gain and loss of *TCP* genes in Asteraceae

NOTUNG software (Chen et al., 2000; Stolzer et al., 2012) was used to perform the gene gain and loss events of TCP gene family in Asteraceae. The genomic data of other Asteraceae species was downloaded from NCBI (https://www.ncbi.nlm.nih.gov/).

A species tree was from the TIMETREE (http://www.timetree.org/) online website (Kumar et al., 2017), while the gene phylogenetic tree was developed utilizing IQ-tree software (Nguyen et al., 2014). The tree species and gene tree were imported into the NOTUNG software and analyzed by the Reconciliation Mode function of NOTUNG, in which *A. thaliana* and *O. sativa* were employed as an outgroup.

### 2.4 Chromosomal localization and duplication events of *OtTCP* genes

The chromosomal locations of *OtTCP* genes were visualized with TBtools software, using the GFF file of the *O. taihangensis* genome database.

To explore the potential evolutionary relationships of *TCP* genes, the collinearity analysis among *A. thaliana*, *O. sativa* and Asteraceae species (*Helianthus annuus*, *Arctium lappa, Cynara cardunculus*, *Cichorium intybus*, *Centaurea solstitialis*, *Erigeron canadensis*, *Lactuca saligna*, *Lactuca virosa*, *Smallanthus sonchifolius*, *Mikania micrantha*, and *Tagetes erecta*) were investigated using the Multiple Collinearity Scan Toolkit (MCScanX) in TBtools (Chen et al., 2020). The genomic data of *A. thaliana* (TAIR 10), *O. sativa* (IRGSP-1.0), *H. annuus* (HanXRQr2.0-SUNRISE), *A. lappa* (ASM2352574v1), *C. cardunculus* (CcrdV1.1), *C. intybus* (ASM2352571v1), *C. solstitialis* (ASM3016916v1), *E. canadensis* (C_canadensis_v1), *L. saligna* (Lactuca_saligna), *L. virosa* (Lvir_assembly_v4), *S. sonchifolius* (ASM2352597v1), *M. micrantha* (ASM936387v1), and *T. erecta* (ASM3086718v1) was downloaded from NCBI (https://www.ncbi.nlm.nih.gov/).

Gene repetition events (such as tandem replication and fragment replication) were performed using MCSCANX in TBtools (Wang et al., 2012; Chen et al., 2020). The TCP protein sequences of these species were aligned using Blastp program in TBtools (Chen et al., 2020), with an e value of 1 × 10^−10^, other parameters set to default values.

The Ka (nonsynonymous substitution per site) and Ks (synonymous substitution per site) (Zhang et al., 2006) between segmental and tandem duplicate gene pairs were calculated by the simple Ka/Ks Calculator in TBtools (Chen et al., 2020). The Ka/Ks value was further utilized to identify the selection mode of *OtTCP* genes.

### 2.5 Secondary and tertiary structures of OtTCP proteins

The secondary and tertiary structures of OtTCP proteins were predicted and modelled using the SOPMA (https://npsa-prabi.ibcp.fr/cgi-bin/npsa_automat.pl?page=npsa_sopma.html) (Geourjon and Deléage, 1995), SWISS MODEL (https://npsa-prabi.ibcp.fr/cgi-bin/npsa_automat.pl?page=npsa_sopma.html) servers (Waterhouse et al., 2018), while the tertiary structures were examined by PyMOL (Rosignoli and Paiardini, 2022) (http://www.pymol.org/pymol).

### 2.6 Cis-acting elements and gene expressions of *OtTCP* genes

The cis-acting elements were predicted using 2000 bp sequences upstream of *OtTCP* genes in PlantCARE (https://bioinformatics.psb.ugent.be/webtools/plantcare/html/) online website (Lescot et al., 2002), the relative results were visualized with GSDS online website 2.0 (Hu et al., 2015).

The transcriptome data (PRJNA400848, PRJNA437359) of *O. taihangensis* under drought treatments were downloaded from NCBI (Gu et al., 2019; Yang et al., 2020). And the transcriptome sequencing data of *O. taihangensis* different tissues under salt stress were from our laboratory. Under 500 mM/L salt treatment, *O. taihangensis* individuals were treated for 0, 6, 24 and 48 h respectively. While under 24 h treatment, the sampled individuals were treated with 0 mM/L, 100 Mm/L, 300 Mm/L and 500 Mm/L salt respectively. Three replicates were set up for each treatment. After treatments, the sampled leaves from the same sites of each individual were frozen in liquid nitrogen for transcriptome sequencing (Han et al., 2024; Ye et al., 2024; Zhang et al., 2024c; Zhou et al., 2024). Based on the above, an expression heatmap of *O. taihangensis* under different treatments was generated using TBtools (Chen et al., 2020).

### 2.7 Expression validation by qRT-PCR

Finally, qRT-PCR was conducted to validate the expression patterns of randomly selected *TCP* genes of *O. taihangensis*. According to Peng et al (Peng et al., 2024), the internal reference genes were selected for evm. TU. Chr8.13443 (Han et al., 2024; Zhang et al., 2024c) and evm. TU. Chr8.39 (Zhou et al., 2024). Three technical replicates were performed for each selected gene. The PCR primers were designed using PRIMER 5.0 software (**Supplementary Table S1**), and the primer efficiency was evaluated from the amplification of three replicates based on Bello et al (Bello et al., 2017).

The qRT-PCR was performed with the UltraSYBR mixture (TaKaRa, Dalian, China) using an ABI7500 RT-PCR system. Reactions were done in 20 μl volume, the following qRT-PCR program was used: the template denaturation at 95°C for 3 min; followed by amplification for 40 cycles with a melting temperature of 95°C for 10s and an annealing temperature of 68°C for 15s. After 40 cycles, the melting curve analysis ranged from 60°C to 95°C, and the amplification efficiency was determined from the slope of the standard curve linear-log of target genes. All relative gene expression levels were calculated using 2^−ΔΔCT^ (Penfield et al., 2001).

## 3 Results

### 3.1 Identification and physicochemical properties of OtTCPs

A total of 14 *TCP* genes with conserved domains were identified in *O. taihangensis*, which were designated *OtTCP1* - *OtTCP14* based on their locations on the chromosomes (**Table 1**).

As shown in **Table 1**, OtTCP proteins varied in their lengths, molecular weights, theoretical isoelectric points, and so on. Sequence analyses revealed that the 14 OtTCP proteins ranged from 241 (OtTCP13) to 426 amino acids (OtTCP5), with average lengths of 358 amino acids. The molecular weights ranged from 26398.93 to 45725.23 Da. For the theoretical pI, significant differences between the OtTCP proteins suggested that they might function under various acidic and basic conditions. The lowest (5.39) and highest (9.74) pI were OtTCP4 and OtTCP14, respectively. Thereinto, there were 7 OtTCP proteins (50%) with pI values of < 7.0, which indicated that they contained an abundance of acidic amino acids. All OtTCP proteins were unstable with a values of over 40 instability index (Guruprasad et al., 1990). More, almost all of the OtTCP proteins were hydrophilic that had a negative grand average of hydropathicity (GRAVY) values. Furthermore, all 14 OtTCP proteins were located within the nucleus.

### 3.2 Phylogenetics, gene structures, and conserved motifs of OtTCPs

**Figure 1:**
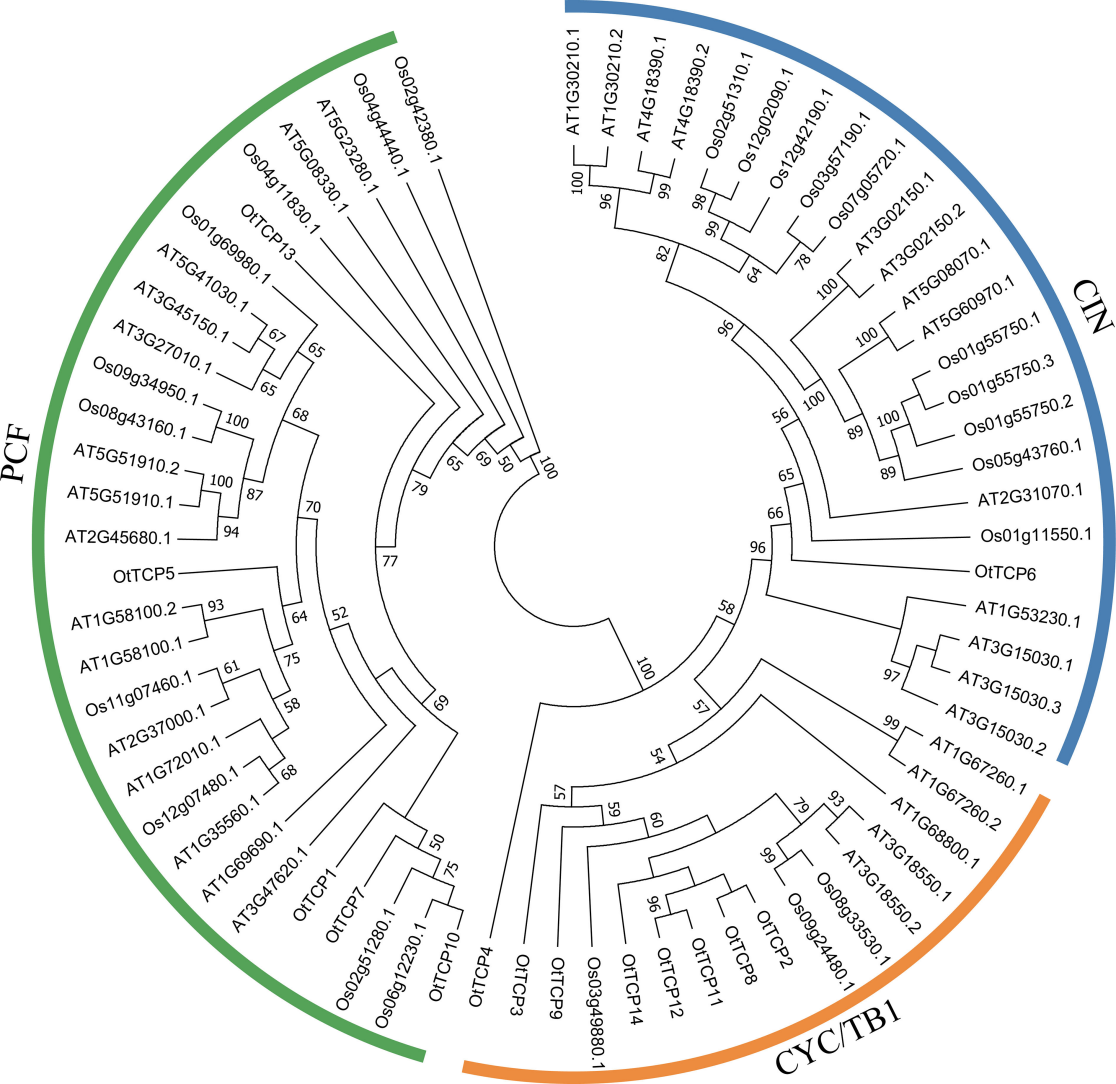
The constructed phylogenetic tree based on the TCP proteins of *O. taihangensis* (Ot), *Arabidopsis thaliana* (At), and *Oryza sativa* (Os). The TCP gene family was mainly divided into two clades: Class I (PCF) and Class II with possessing two subclades (CYC/TB1 and CIN).

A total of 70 complete protein sequences, including 33 AtTCPs (*A. thaliana*), 23 OsTCPs (*O. sativa*), and 14 OtTCPs, were used in the phylogenetic analysis. Based on a phylogenetic tree, all analyzed *TCP* genes were segregated into two main classes: Class I (PCF) and Class II (CIN and CYC/TB1) (**Figure 1**). Class I was the largest group, which contained five OtTCPs, ten OsTCPs, and fifteen AtTCPs. While class II included eight OtTCPs, three OsTCPs, and five AtTCPs. Interestingly, the CIN group in Class II contained only one OtTCP member (OtTCP6).

**Figure 2:**
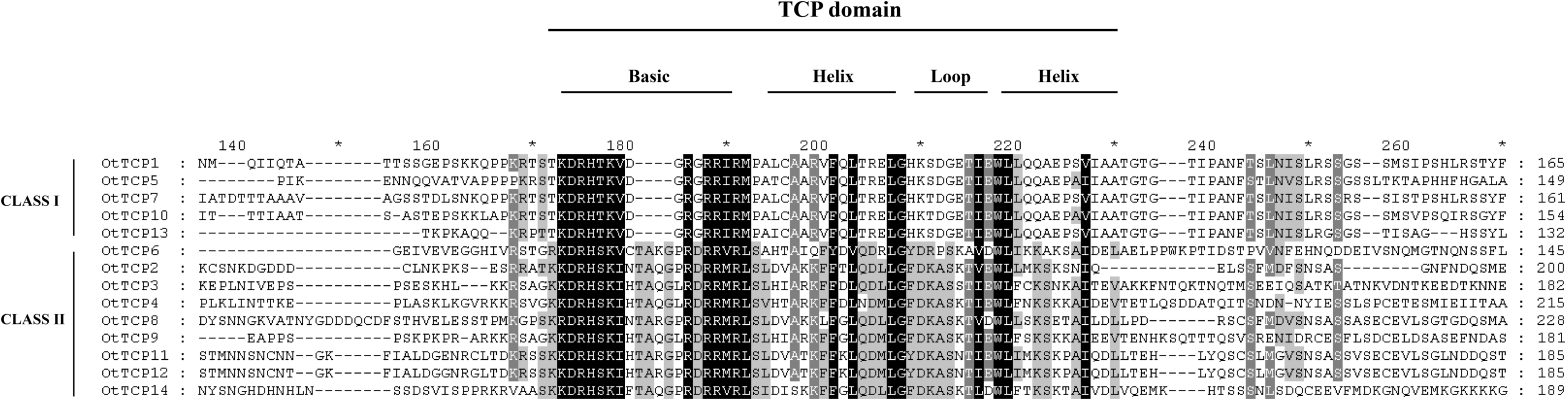
Multiple sequence alignment of OtTCP protein was divided into an alkaline region basic and Helix I–Loop–Helix II.

Conserved domain sequence alignment analysis was conducted to gain further insights into the evolutionary relationships and structural characteristics of *OtTCP* genes. The results (**Figure 2**) revealed that all 14 OtTCPs possessed a conserved domain of 60 amino acid residues. This conserved domain included a primary region at the N-terminus and a HLH (helix–loop–helix) motif at the C-terminus, which was consistent with the TCPs’ structure observed in other plant species (Liu et al., 2022a; Jiang et al., 2023; Wu et al., 2023). Notably, the primary regions of Class I of OtTCPs contained four fewer amino acid residues than that of Class II (**Figure 2**).

**Figure 3:**
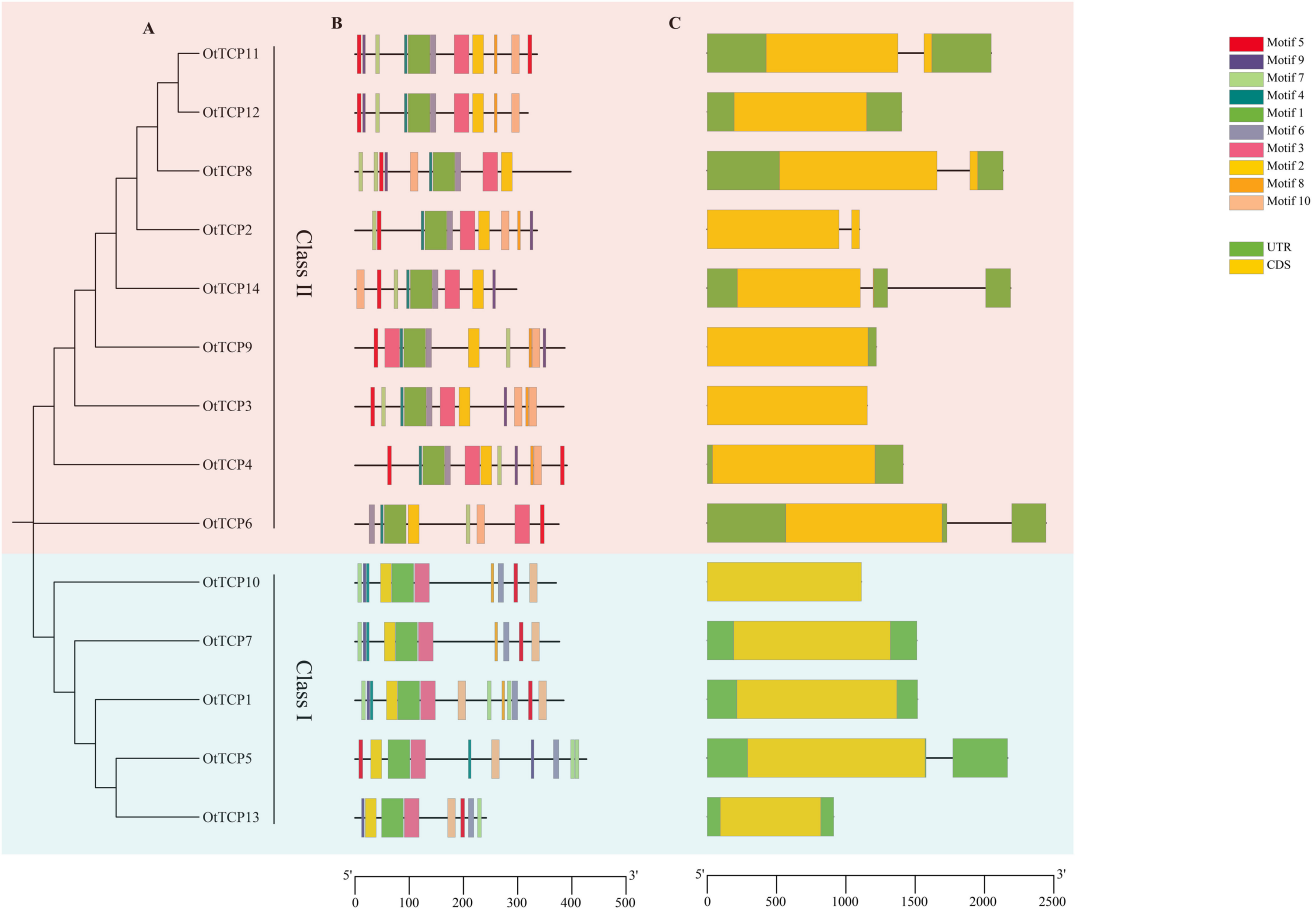
**(A)** Phylogenetic tree of the OtTCPs with two classes. **(B)** Conserved motifs of the OtTCP proteins. Different color represented different motif. **(C)** Exon-intron organization of *OtTCP* genes. Yellow boxes represented exons (CDS), green boxes represented UTR, and grey lines represented introns. The scale was the sizes of exon or intron.

Regarding the OtTCPs exons and introns, six genes (42.8%) contained introns, while the remaining 8 *TCP* genes (57.2%) had none. The *OtTCP* genes of Class I possessed more introns than did Class II. Of the *OtTCP* genes, five possessed a single intron, whereas only one gene (*OtTCP14*) had two (**Figure 3C**).

Ten conserved motifs were identified and designated as motifs 1 - 10. In the CYC/TB1 group of Class II, most genes contained ten motifs except for OtTCP14. The CIN group of Class II did not include motifs 8 and 9, while in the PCF group of Class I, three OtTCPs contained all ten motifs. Further, OtTCP5 did not contain motif 8 and OtTCP13 did not include motifs 4 and 8. Overall, the genetic structures and conserved motifs of most OtTCPs within the same class were similar.

### 3.3 Chromosomal location, collinearity, and evolution of OtTCPs

**Figure 4:**
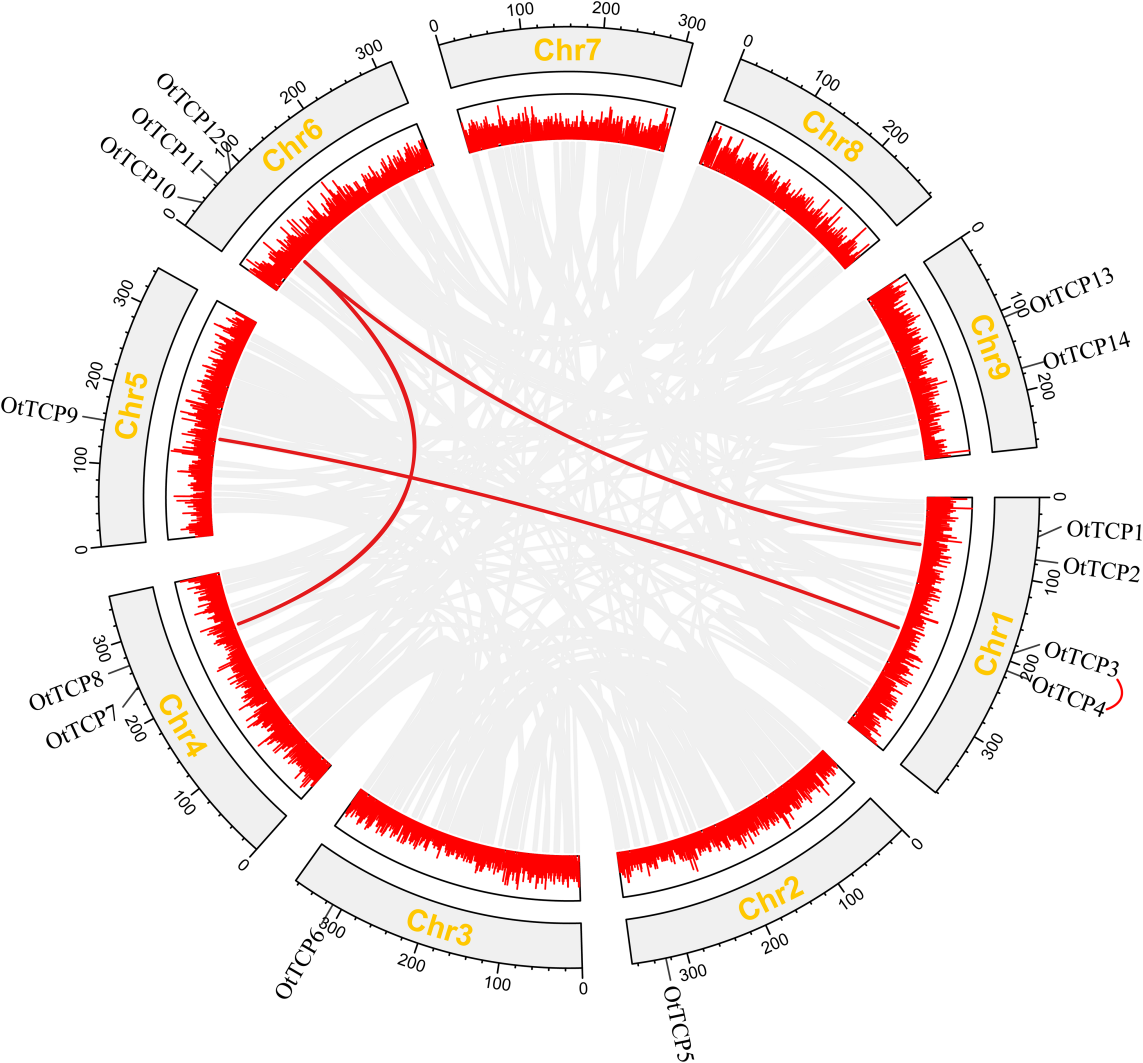
Replication events of *OtTCP* genes. Gray lines represented the duplicated genes, while the red lines represented the segmental duplicate *TCP* gene pairs. Additionally, the red lines connecting genes outside the chromosome represented the tandem duplicated pairs. Box with red line graph showed the gene densities. Gray rectangles represented the chromosomes, the corresponding names displayed externally for each chromosome.

The locations of *OtTCP* genes on chromosomes were relatively dispersed (**Figure 4**). Chromosome 1 contained the most *OtTCP* genes (4 genes, ~28.6%), followed by chromosome 6 (3 genes, ~21%), while chromosomes 2, 3, and 5 had the least (1 gene, ~7%). Chromosomes 4 and 9 held the same number of *OtTCP* genes (2 each, ~14%), while chromosomes 7 and 8 had no *OtTCP* genes.

The duplication events of *OtTCP* genes were analyzed, showing that only one tandem repeat gene pair (*OtTCP3*-*OtTCP4*) was found on chromosome 1. Three segmental duplication events (*OtTCP8*-*OtTCP11*, *OtTCP2*-*OtTCP11* and *OtTCP4*-*OtTCP9*) were detected to be scattered across four chromosomes. These results suggested that tandem and segmental duplication events may play key roles in the OtTCP gene family.

The substitution Ka/Ks ratio was used to elucidate OtTCPs evolutionary processes and selection pressures, where a Ka/Ks value of 1 indicated neutral selection, < 1 denoted purification selection, and Ka/Ks > 1 signified positive selection. The Ka/Ks value for tandem duplication was 0.31778, while that for segmental duplication varied from 0.2681 to 0.3804 with a mean value 0.3292 (**Supplementary Table S2**). The Ka/Ks value for all duplication events was < 1, which implied that *OtTCP* genes evolved under the effects of purifying selection.

**Figure 5:**
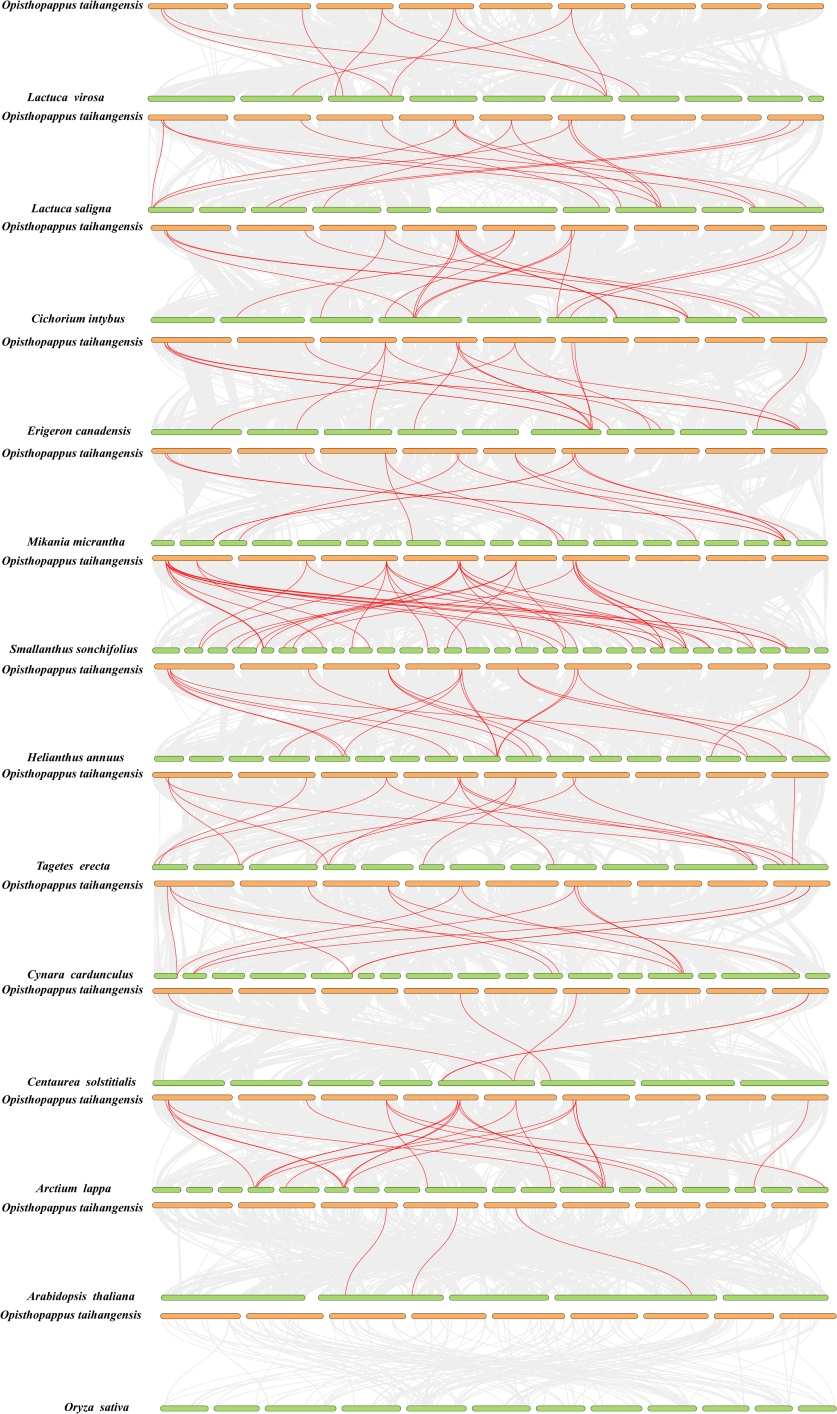
The collinearity analysis among *A. thaliana*, *O. sativa* and 11 Asteraceae species. The red line represents *TCP* collinear gene pairs in *O. taihangensis* and other genomes.

To further illustrate the potential evolutionary relationships of the OtTCP gene family, the comparative collinearity relationships were identified between *O. taihangensis* and the other 13 species (**Figure 5**).

To further illustrate the potential evolutionary relationships of the OtTCP gene family, the comparative collinearity relationships were identified between *O. taihangensis* and the other 13 species (**Figure 5**). The collinear revealed that there were 3 collinear gene pairs between *O. taihangensis* and *A. thaliana*, no collinear genes were found in *O. sativa*. In Asteraceae, 9, 15, 18, 17, 13, 38, 22, 17, 14, 5, and 22 of collinear gene pairs were identified in *O. taihangensis* with *L. virosa*, *L. saligna*, *C. intybus*, *E. canadensis*, *M. micrantha*, *S. sonchifolius*, *H. annuus*, *T. erecta*, *C. cardunculus*, *C. solstitialis*, and *A. lappa*, respectively. The large number of collinear pairs between *O. taihangensis* and *S. sonchifolius* indicated a closely relationship among them. Notably, some *OtTCP* genes were found to have at least three collinear pairs (particularly between *O. taihangensis* and *H. annuus*), such as *OtTCP2*, *OtTCP3*, *OtTCP6*, and *OtTCP8* (**Supplementary Table S3**).

**Figure 6:**
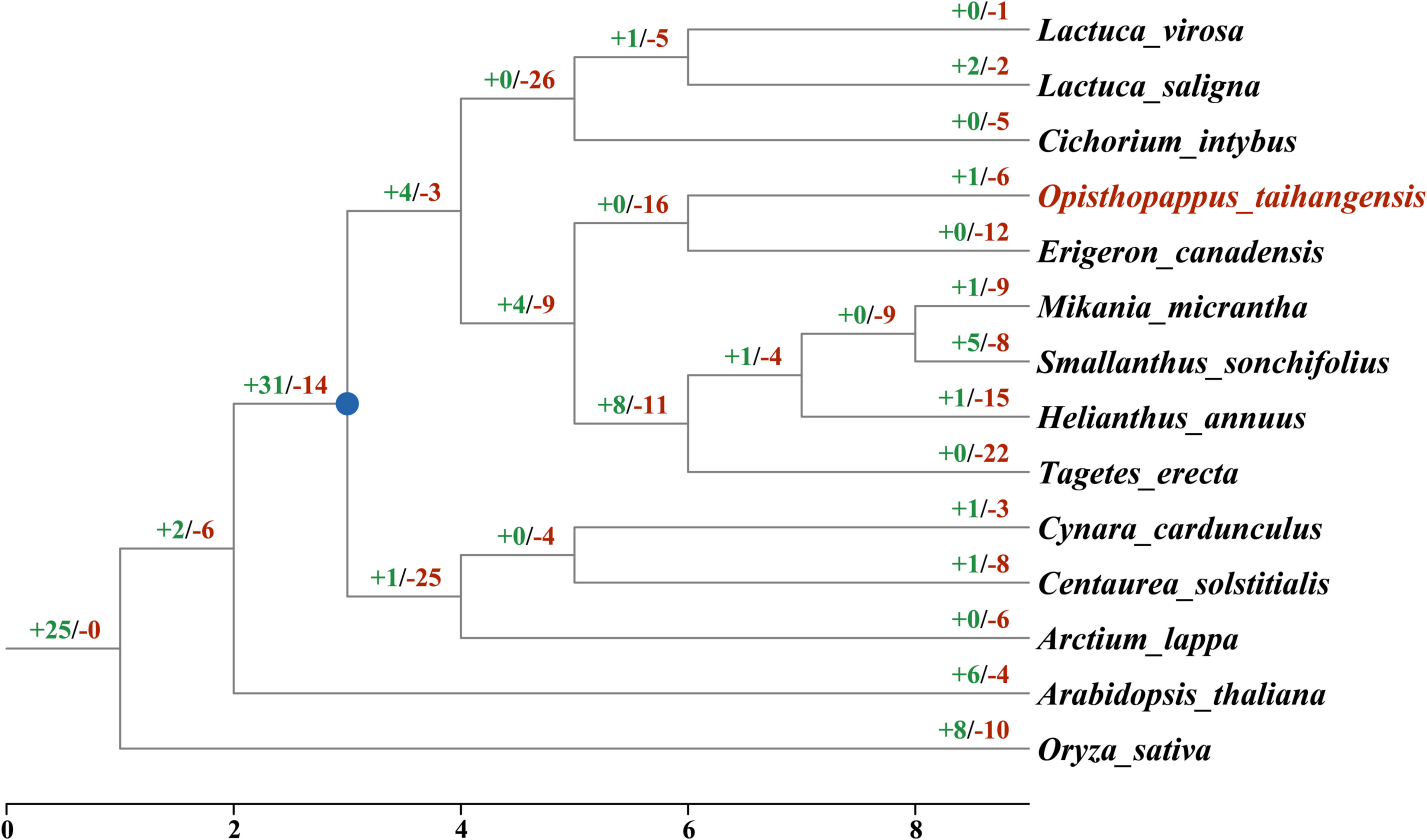
The gain and loss events of *TCP* genes in the Asteraceae. The numbers at the branch nodes represented the gained and lost genes. The blue nodes represented the possible common ancestor of the Asteraceae.

A gene gain and loss analysis revealed that *TCP* genes underwent a dramatic dynamic change in Asteraceae (**Figures 6**; **Supplementary Figure S1**), as 103 gain and 243 loss events occurred (**Figure 6**). Differentiation from the outgroups, the common ancestor of Asteraceae TCPs underwent duplication (+31). Subsequently, loss events during Asteraceae evolution occurred that resulting in the TCP gene family continuously contracted. The most loss occurred in *T. erecta* (-22), followed *H. annuus* (-15) and *E. canadensis* (-12), while the lowest in *L.virosa* (-1), *L. saligna* (-2) and *C. cardunculus* (-3). For *O. taihangensis, TCP* genes underwent one duplication events and six loss events.

### 3.4 Secondary and tertiary structures of OtTCP proteins

The results of investigations into the secondary structures of OtTCP proteins (**Supplementary Table S4**) indicated that they were primarily comprised of α-helices (12.27%–34.03%), extended strands (8.36%–16.60%), β-turns (1.19%–9.13%), and random coils (2.05%–73.33%). The tertiary structures contained α-helices, β-turns, and random coil structures (**Supplementary Figure S2**), which translated to distinct OtTCP protein conformations and implied their functional differentiation.

### 3.5 Cis-elements of *OtTCP* genes

**Figure 7:**
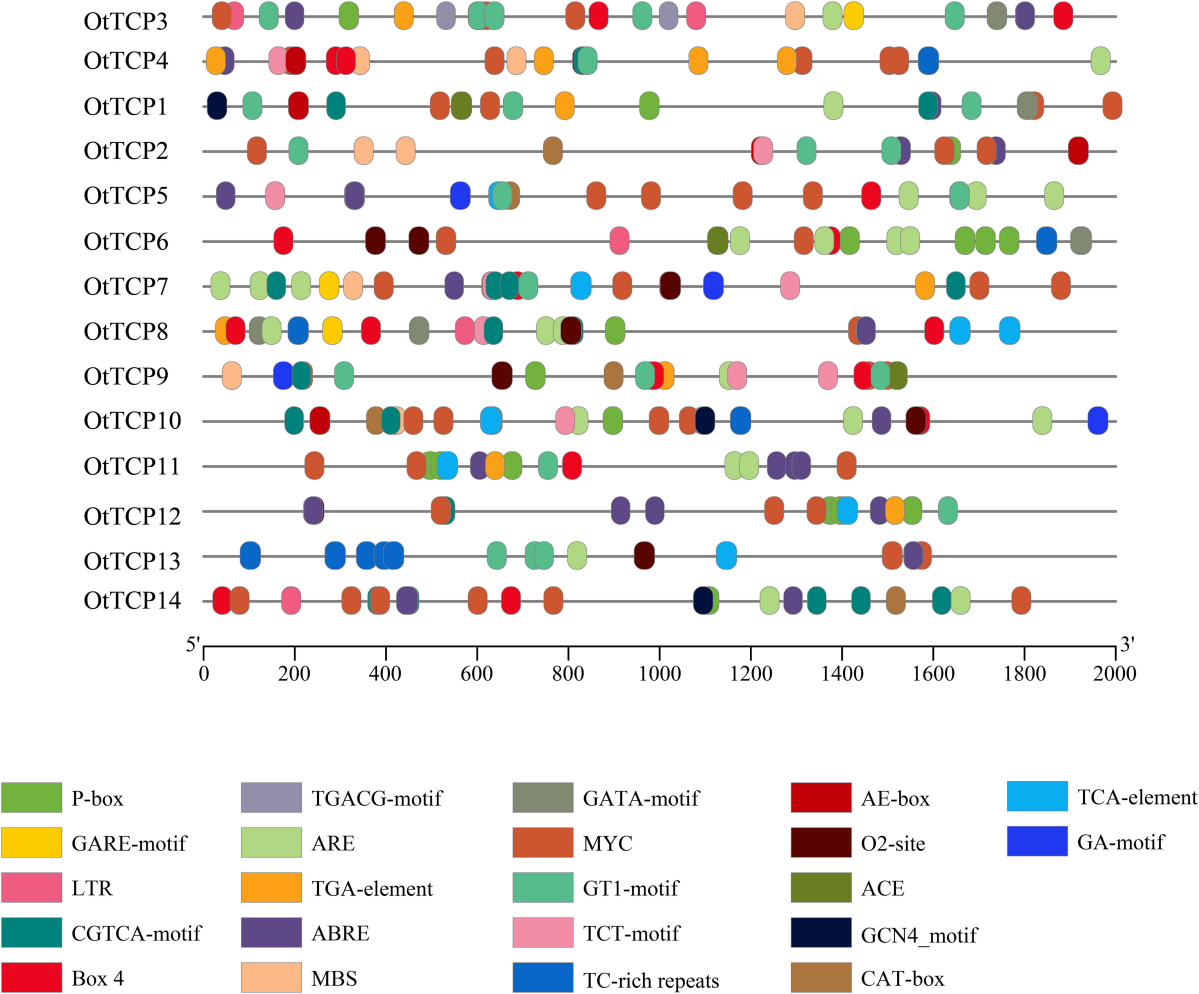
Cis-acting elements in the OtTCP promoter regions. Different colors represented various elements.

In total, 305 cis-acting elements attributed to 22 types were identified in *OtTCP* genes (**Figure 7**; **Supplementary Table S5**). These elements were segregated into four categories (light response, growth and development, hormone response, and stress-related cis-acting elements).

All *OtTCP* genes had light responses. However, the types and quantities of each *OtTCP* gene varied, which suggested that light signals may positively impact transcriptional regulation processes. Twelve *OtTCP* genes contained cis-acting elements related to growth and development, including the CGN4-motif, CAT-box, and O2-site. Hormone-responsive elements, such as ABRE (ABA-responsive element), TGA elements, CGTCA motifs, and TGACG motifs (elements involved in MeJA responsiveness), and Gibberellic acid-responsive elements (GAREs), were also screened. ABA-responsive elements (ABREs) were identified in 12 (86%) *OtTCP* genes (save for *OtTCP6* and *OtTCP9*). Further, stress-related cis-regulatory elements including MBS (drought-induced response element), LTR (low-temperature response element), ARE (anaerobically induced response element), and TC-rich (defense and stress response element), were identified in the promoter regions of 14 *OtTCP* genes.

### 3.6 Expressions of *OtTCP* genes in response to abiotic stress

**Figure 8:**
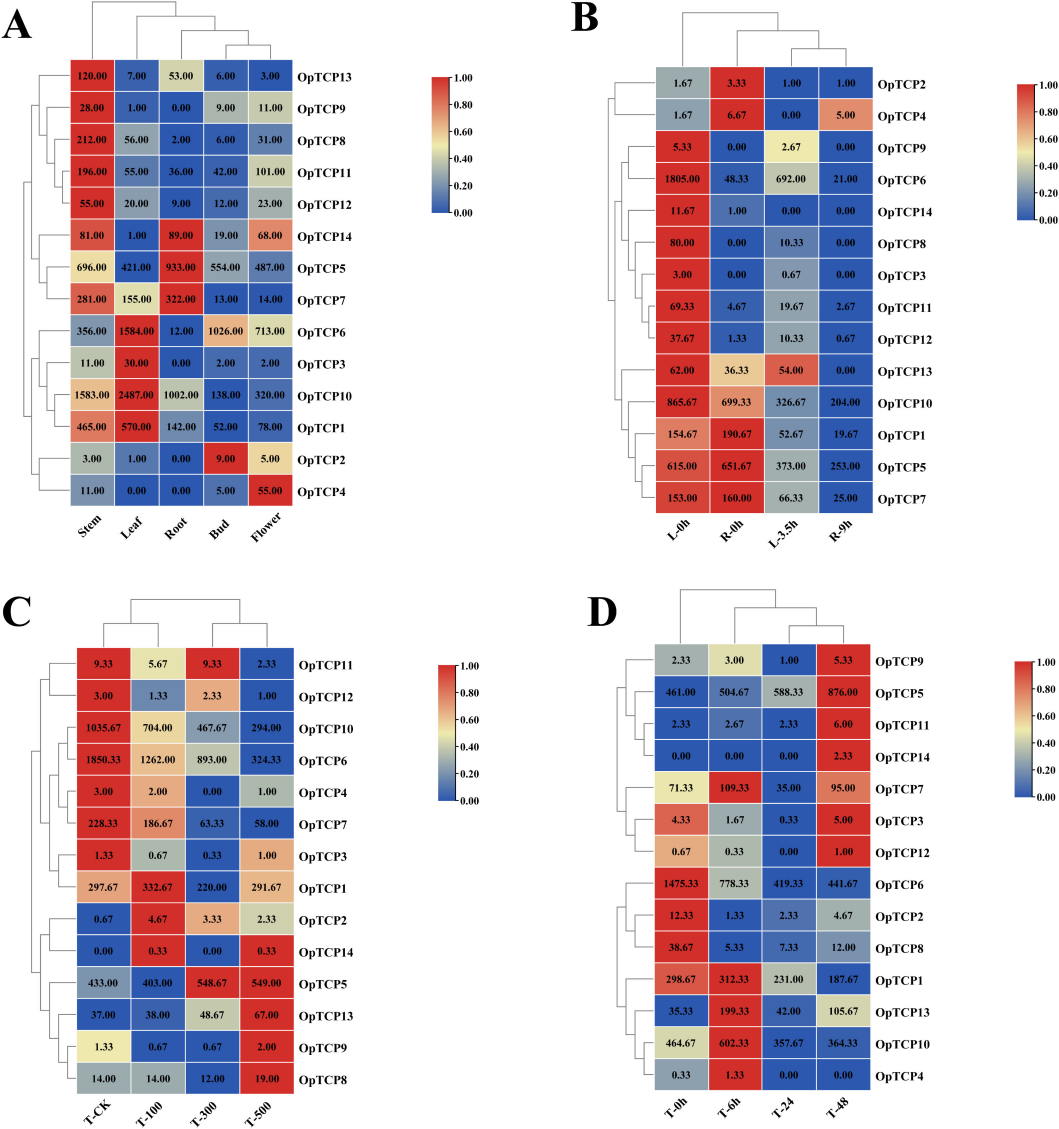
Expression patterns of OtTCPs. **(A)** Expression profiles of the OtTCPs in different tissues. **(B)** Leaves treated with 20% PEG6000 for 3.5 hours or untreated (0 hours); Roots treated with 20% PEG6000 for 9 hours or untreated (0 hours). **(C)** 100 Mm/L, 300 Mm/L, and 500 Mm/L mixed salt solution for 24h treatment. **(D)** 500 Mm/L mixed salt solution in leaves for 6h, 24h, and 48h treatment. The different colored boxes indicated the different log2 (FPKM) values, the red blocks indicated high relative expression levels and blue blocks indicated low relative expression levels.

Using the download and our previous transcriptomic datasets (Ye et al., 2024), we analyzed the expressions of 14 *OtTCP* genes in different *O. taihangensis* tissues, including stems, leaves, roots, buds, and flowers (Gu et al., 2019; Yang et al., 2020) (**Figure 8A**; **Supplementary Table S6**). Tissue-specific expressions were predominantly observed for 8 *OtTCP* genes (*OtTCP1*, *OtTCP7*, *OtTCP8*, *OtTCP9*, *OtTCP11*, *OtTCP12*, *OtTCP13*, and *OtTCP14*) in stems; 4 *OtTCP* genes (*OtTCP1*, *OtTCP3*, *OtTCP6*, and *OtTCP10*) overrepresented in leaves; 3 *OtTCP* genes (*OtTCP5*, *OtTCP7*, and *OtTCP14*) highly expressed in roots; *OtTCP2* mainly expressed in buds, and *OtTCP4* primarily expressed in flowers.

To further explore the roles of these *OtTCP* genes under drought and salt stress, we compared the expression patterns across various treatments (**Figure 8**; **Supplementary Tables S7**, **S8**). The expression levels of *OtTCP* genes were diverse, with 12 genes being highly expressed in leaves, and seven being highly expressed in roots at 0h under a 20% PEG6000 treatment. All genes showed a downward trend when subjected to different levels of drought stress. Overall, the *TCP* genes showed high expression levels in leaves, but not in roots, which presented tissue-specific expression patterns.

The expressions of most *OtTCP* genes were altered under increasing salt concentrations (**Figures 8C, D**). Under the 100 mM and 300 mM treatments, the expressions of most genes were rapidly induced in the early stages (e.g., *OtTCP1*, *OtTCP2*, *OtTCP3*, *OtTCP4*, *OtTCP5*, *OtTCP7*, and *OtTCP14*). The expressions of *OtTCP5*, *OtTCP8*, *OtTCP9*, *OtTCP13*, and *OtTCP14* peaked at 500 mM/L. Meanwhile, half of the *OtTCP* gene expressions increased over time gradients (**Figure 8D**).

### 3.7 qRT-PCR quantitative verification

**Figure 9:**
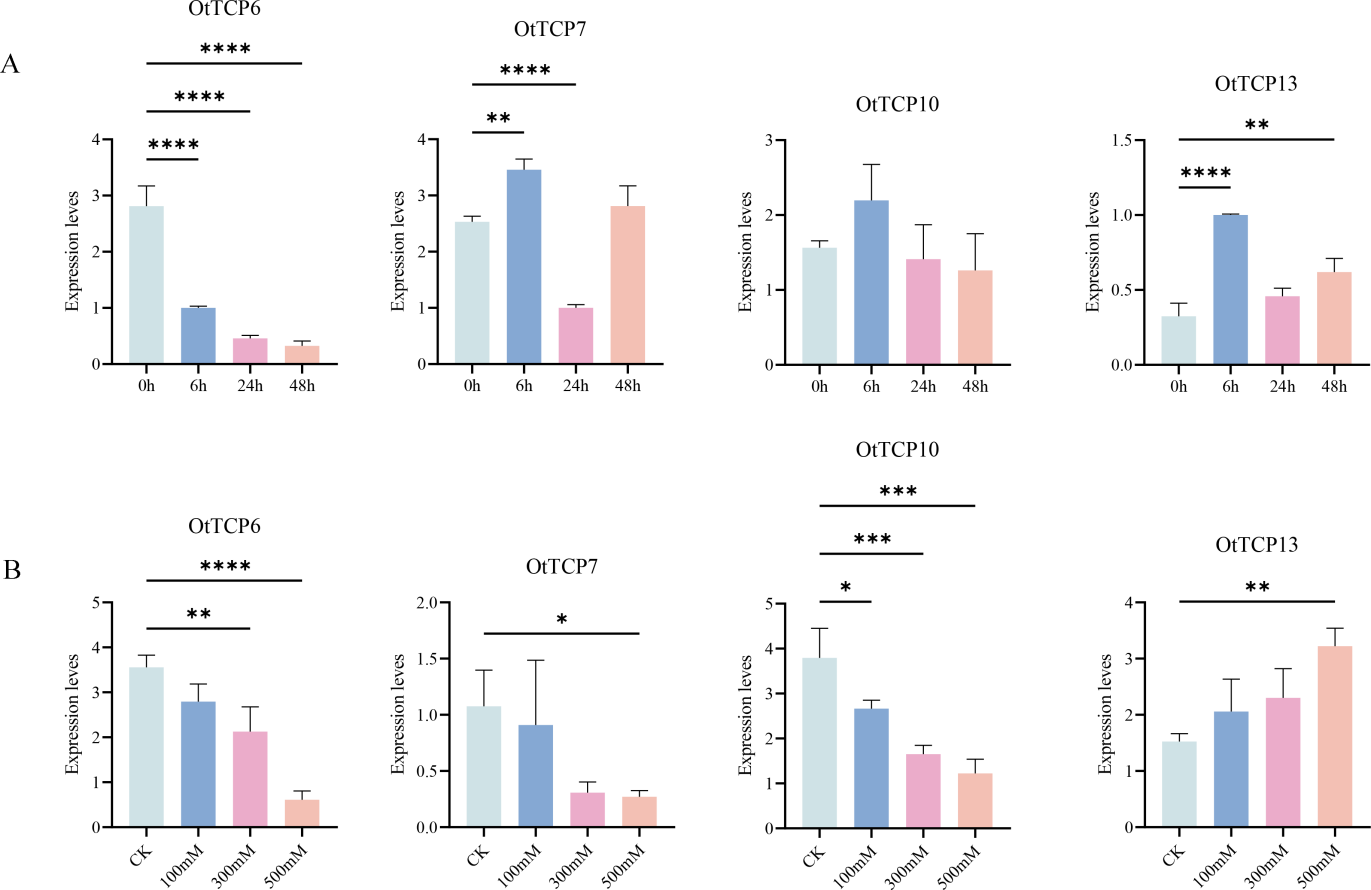
Expression patterns of *OtTCP* genes under qRT-PCR. **(A)** represented the treatment of 0h, 6h, 24h, and 48h of 500 Mm/L salt stress. **(B)** represented CK, 100 Mm/L, 300 Mm/L, and 500 Mm/L salt stress for 24h treatment. Vertical bars represented the mean ± SD of three biological replicates. Statistical significance was determined using one-way ANOVA (**** p < 0.0001 *** p < 0.001 ** p < 0.01* p < 0.05).

The qRT-PCR showed the relative genes were significant induced or inhibited under various salt treatments, with their expression levels consistent with the previous results. These verified the precision of our analyses (**Figure 9**).

## 4 Discussion

Multiple studies have established that *TCP* genes play a widespread role in diverse physiological and biological processes, encompassing plant growth and abiotic stress responses (Huo et al., 2019; Liu et al., 2022a; Panzade et al., 2024). In this study, we performed the whole genome and transcriptomic data to explore the TCP gene family in *O. taihangensis*.

### 4.1 Gene structures and characteristics of OtTCPs

A total of 14 identified *TCP* genes (OtTCP) in *O. taihangensis* were classified into two main clades (Class I and Class II) and three subfamilies (PCF, CIN, CYC/TB1). Within each subfamily, the *TCP* gene members were from *O. taihangensis*, *A. thaliana*, and *O. sativa*. This suggested that these genes originated from the common ancestors, which was consistent with preceding studies on other species (Jiang et al., 2023).

Gene structures and conserved motifs provide clues for the prediction of the evolution of genes and their corresponding proteins (Cao et al., 2019). The *TCP* genes in *O. taihangensis* lacked introns or had only single or two introns (**Figure 3**). This structural feature was also found in *Camellia sinensis* (Shang et al., 2022), *Cymbidium goeringii* (Liu et al., 2022b), and *Dactylis glomerata* (Wang et al., 2023a). With fewer introns, genes can rapidly generate more proteins and quickly respond to abiotic stresses (Ma et al., 2021). For *OtTCP* genes, the lack of introns might be a strategy for responding to abiotic stresses.

Previous studies revealed that the CIN clade was relatively ancient in the TCP gene family. In this study, the CIN clade contained only one gene (*OtTCP6*) that possessed one intron (**Figure 3**). *OtTCP* genes gradually lose introns during evolution, which might be due to inversion or homologous recombination with intron-containing genes (Wu et al., 2005). *OtTCP1*, *OtTCP4*, *OtTCP7*, *OtTCP10*, and *OtTCP13* had no introns; thus, they could rapidly express under abiotic stress (**Figures 7C, D**). Smaller genes (such as *OtTCP14*) that contained more introns might be involved in biological processes such as mRNA output and alternative splicing, which could modify their functionalities to a certain extent (Roy and Gilbert, 2006).

Meanwhile, in terms of structure, ancient *OtTCP6* contained no motifs 8 or 9. This suggested that certain members of Class I (PCF) and Class II (CYC/TB1) eventually experienced increases in motifs 8 and 9. These structural changes may either support original functionality or induce increased functional diversity.

### 4.2 Evolution of *TCPs*

In contrast to other plants, the number of *TCP* genes in *O. taihangensis* was lower than that in *Chrysanthemum lavandulifolium* (39) (Wu et al., 2023), *Chrysanthemum nankingense* (23) (Yu et al., 2022b), *A. thaliana* (33) (Yu et al., 2022a) and *O. sativa* (23) (Li et al., 2017).

Generally, there were 14 to 38 TCPs found in angiosperms (Liu et al., 2019). Moreover, the *TCP* gene number was increased with the evolution of species from early-diverging to later-diverging (Martín-Trillo and Cubas, 2010; Shang et al., 2022). For example, *Amborella trichopoda* (belonging to *Amborella* of *Amborellaceae*) was a species of basic angiosperm group and had 15 *TCP* genes. Some eudicots, such as *Aquilegia coerulea* (14), *Citrus sinensis* (15), *Eucalyptus grandis* (16), and *Vitis vinifera* (15), also had less *TCP* genes (Liu et al., 2019). In Asteraceae, *Opisthopappus* genus was regarded as a relative close ancestral group of *Ajania* (Zhao, 2007). *O. taihangensis* possessing 14 *TCP* genes may be related with its phylogenic position (Shen et al., 2021).

Through chromosome localization analysis, it was found that *OtTCP* genes were unevenly distributed across nine chromosomes in the *O. taihangensis* genome. The uneven distribution of genes in genomic chromosomes was closely related to extensive gene loss, which is pervasive in angiosperm (Sun et al., 2023).

As know, whole genome duplication (WGD) is one of the most important driving forces for genome evolution (Magadum et al., 2013). Large number of duplicated genes would be produced after WGD (Chen et al., 2023). Here, we identified *OtTCP* genes experienced by tandem and segmental duplication (**Figure 4**). In our other study (unpulished), *O. taihangensis* genome was detected undergone WGD event at 59 Mya. However, more loss (-6) and less gain (+1) events of *OtTCP* genes occurred during the evolution (**Figures 4** and **Supplementary Figure S1**). Some studies showed that the frequency of gene loss is up to three times higher than the rate of gene gain (Koskiniemi et al., 2012; Puigbò et al., 2014; Nelson-Sathi et al., 2015). After WGD, some functionally important gene copies can be retained, whereas some functionally redundant gene copies would be lost or pseudogenized (Duan et al., 2014; Liang et al., 2016). For *O. taihangensis TCP* genes, the loss events should be post whole genome duplication, and only keep some important copies (Li et al., 2024). Based on Zhang et al. (2024a), *CYC2* genes of TCP family were experienced the duplications that predated their gains during the evolution of florets and floral symmetry in Asteraceae. More, the loss of *CYC2d* were found in the formation of ligulate florets (Chen et al., 2018; Zhang et al., 2024a). These may support our results at a certain extent.

Gene loss can contribute to species’ adaptive evolution, particularly in response to environmental challenges (Albalat and Cañestro, 2016). Under selection, positive selective pressure facilitates gene expansion or functional differentiation, whereas purifying selective pressure often renders more conservative genes (Song and Nan, 2014). Indeed, purifying selection occurred during the evolution of *OtTCP* genes (with Ka/Ks values consistently < 1). This selective pressure may have ultimately led to the contraction or loss of OtTCPs (Wu et al., 2022). The gene loss in the OtTCP family might be an adaptive strategy for *O. taihangensis* on the cliff habitats.

On the other hand, the high collinearity (38 syntenic blocks) occurred between *O. taihangensis* (one member of Asterodae) and *S. sonchifolius* (one member of Helianthodae) in the studied Asteraceae species (**Figure 5**). It indicated that these genes located in corresponding syntenic blocks occurred before the divergence of *O. taihangensis* and *S. sonchifolius*. Asterodae and Helianthodae both were the members of Asteroideae and diverged about 57.71 Mya after ancient WGD event (Zhang et al., 2024a). High collinearity among the two species should be happened before 57.71 Mya.

### 4.3 Role of OtTCPs under abiotic stress

As pivotal molecular switches, cis-regulatory elements participate in the transcriptional regulation of genes and control a variety of biological processes (Huang et al., 2021). OtTCPs were found that enriched with cis-regulatory elements associated with growth and development, hormone signaling, and stress responses (**Figure 7**).

All *TCP* genes of *O. taihangensis* possessed photonically responsive elements, indicating that *OtTCP* genes responded to light for the regulation of growth and development in *O. taihangensis*, which aligned with the results of *C. goeringii* (Liu et al., 2022b). A dozen of 14 *OtTCP* genes contained ABRE cis-regulatory elements associated with ABA responsiveness. ABRE-binding protein/ABRE-binding factor (AREB/ABF) can positively regulate the plant responses and enhance tolerance (Fujita et al., 2013; Yang et al., 2024), while the ABA signaling pathway is crucial for abiotic stress resistance. Plants challenged by water deficits, salinity, cold, or pathogen attacks induce the accumulation of ABA, which translates to gene expression via ABRE cis-acting elements to defend against these stresses (Dar et al., 2017). *OsTCP19* gene from rice, which activated by salt, drought, and cold stresses, enhances ABA signal transduction by promoting the expression of ABA INSENSITIVE4, which interacts directly with relative encoded proteins (Tatematsu et al., 2008; Rueda-Romero et al., 2012). The *TCP10* gene of Moso bamboo positively regulates early tolerance by regulating the ABA signaling pathway, which negatively regulates lateral root growth via the methyl jasmonate (Me-JA)-mediated signaling pathway (Xu et al., 2022). In *A. thaliana*, the *TCP14* gene interacts with the DNA BINDING WITH ONE FINGER 6 transcription factor, inhibiting the activation of the ABA biosynthetic gene ABA DEFICIENT1 and other ABA-related stress genes, and then promoting the germination of *Arabidopsis* seeds (Tatematsu et al., 2008; Rueda-Romero et al., 2012).

Conversely, MBS (MYB binding site) is renowned for its key roles in stress signaling transduction and drought stress responses (Guo et al., 2023). TC-rich repeats are involved in defense and stress responses, while LTR elements engage low temperature stress responses. Six *OtTCP* genes (*OtTCP2*, *OtTCP3*, *OtTCP4*, *OtTCP7*, *OtTCP9*, and *OtTCP10*), five *OtTCP* genes (*OtTCP4*, *OtTCP6*, *OtTCP8*, *OtTCP10*, and *OtTCP13*), and four *OtTCP* genes (*OtTCP3*, *OtTCP6*, *OtTCP8*, and *OtTCP14*) contained MBS, TC-rich, and LTR cis-regulatory elements, respectively. This indicated that OtTCPs might utilize differential regulatory pathways to counter abiotic stresses.

It is widely recognized that the expression profiles of genes are intimately linked with their functionalities to a large extent. *TCP* genes in *O. taihangensis* exhibit significantly different expression patterns in different tissues and treatments (**Figure 7**). Generally, CYC members in TCP family regulate branching, such as *TB1* in maize and *BRC1* in *Arabidopsis* (Cubas et al., 1999; Aguilar-Martínez et al., 2007); and some involved in flower development (Balsemão-Pires et al., 2013). *OtTCP2*, *3*, *4*, *8*, *9*, *11*, *12*, *14* all were the genes of CYC clade. *OtTCP2* up-expressed in bud*, OtTCP3* in leaf, *OtTCP4* in flower, *OtTCP8*, *9*, *11*, *12* in stem, and *OtTCP14* in stem, root and flower (**Figure 8A**). The members in PCF clade of TCP family Class I also are involved in plant development (Liu et al., 2019). *OtTCP1*, *5*, *7*, *10* and *13* (PCF genes) mainly expressed in stem, root and leaf. Recently, PCF genes were demonstrated to participate in abiotic stresses (Liu et al., 2019). *OtPCF* genes high expressed under different salt treatments in this study (**Figures 8C, D**). *OtTCP* genes expression profiles indicated that their diverse functions, which may play important roles in the growth and development of *O. taihangensis*.

Additionally, some pairs of duplicate genes revealed similar or distinct expression patterns (Zhang et al., 2024b). For example, *OtTCP8* and *OtTCP11* exhibited a negative expression trend under drought treatments. However, under salt stress, *OtTCP11* showed an upward trend, while *OtTCP8* showed the converse. Duplicate genes responded to different stresses through functional diversity. Gene replication can drive the development of new biological functions, which was supported by the tertiary structures of *OtTCP* genes (**Supplementary Figure S2**).

Within different tissues and under salt stress and drought, *OtTCP4*, *9* and *11* presented a relative wide expression (such as *OtTCP4* up-expressed in flower, R-9h, T-100 and T-6h, **Figure 9**). These three genes all were the member of CYC clade and had no or one intron, which contained ABRE and/or MYC/MYB cis-acting elements with the capacity to rapidly respond to stressors. Thus, *OtTCP4*, *9* and *11* could be considered as the candidates for the development, growth and responding to stresses of *O. taihangensis*, although further detailed research is necessary.

## 5 Conclusion

In *O. taihangensis*, 14 *TCP* genes were identified. Compared with other species, relative less *TCP* genes might be accsioated with its ancestral phylogenic position. The *OtTCP* gene family mainly underwent gene loss events after duplication, which could induce adaptive genetic changes. When challenged the stressors, those OtTCPs that lack introns can quickly respond primarily through different cis-regulatory elements. More, *OtTCP* genes exhibit different expression patterns in different tissues and treatments. Thereinto, *OtTCP4*, *9* and *11* could be recognized as important candidates for *O. taihangensis* with a wide expression model. These data may provide clues for the further exploration of the potential resistance mechanisms of *O. taihangensis* in the cliff environments of the Taihang Mountains.

## Data Availability

The data presented in this study are available in the article and **Supplementary Materials**. The transcriptome data of *O. taihangensis* under drought treatment were downloaded from the NCBI with the accession number PRJNA400848 (leaf tissues, 20% PEG6000 treatment for 0 h and 3.5 h with three replicates), PRJNA437359 (root tissues, 20% PEG6000 treatment for 0 h and 9 h with three replicates). The specimens of *O. taihangensis* are stored in the Herbarium of Shanxi Normal University, with the storage number SNUP20230988.
