# Supplementary material for: Identification and expression responses of TCP gene family in Opisthopappus taihangensis under abiotic stress
Source: Front Plant Sci. 2025 Mar 6;16:1499244. doi: 10.3389/fpls.2025.1499244 (PMC11922953; doi:10.3389/fpls.2025.1499244)
Supplement: Supplementary file 1 [file DataSheet1.pdf]

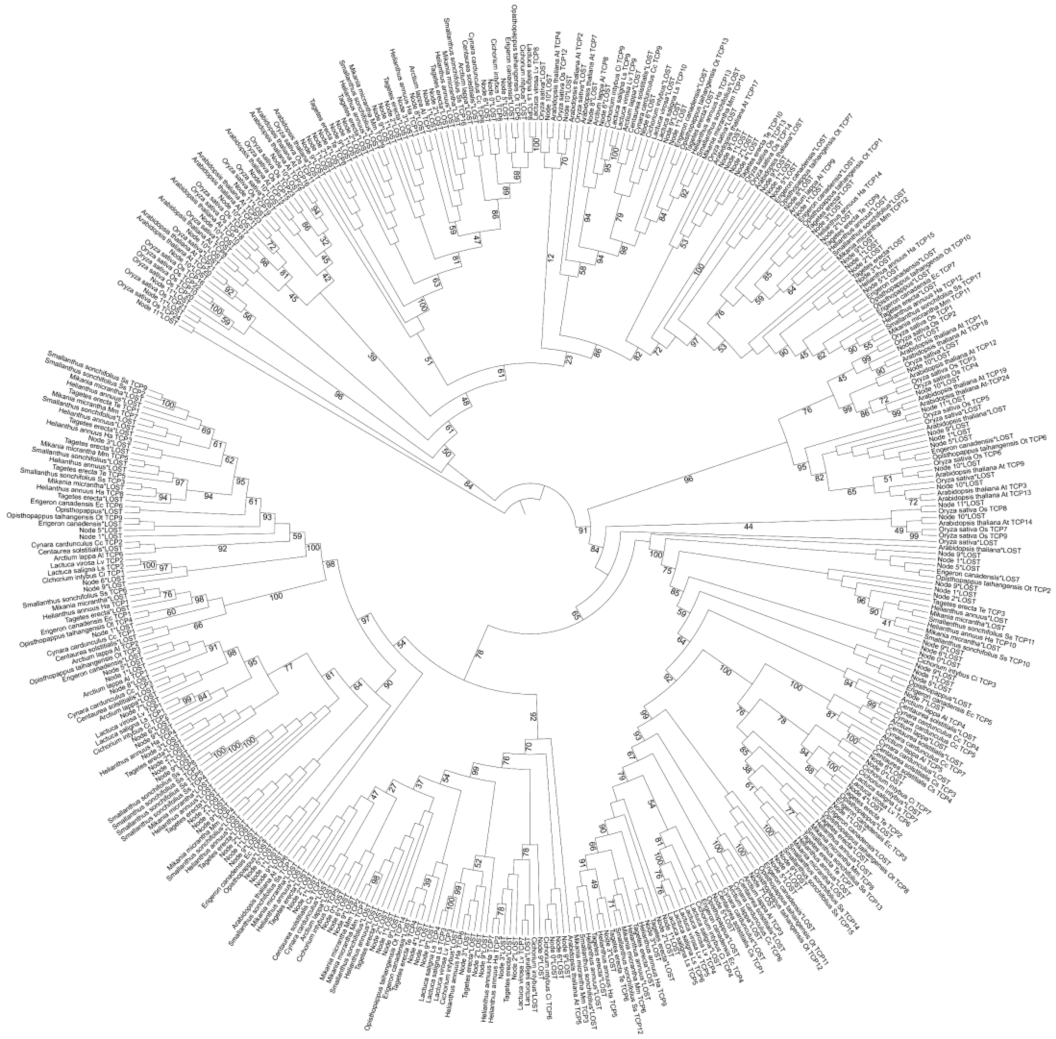

Figure S1: The reconstruction of the gene tree to show the gene gain and lost of TCPs in Asteraceae.The number around the nodes represented bootstrap value with1000 times replications.

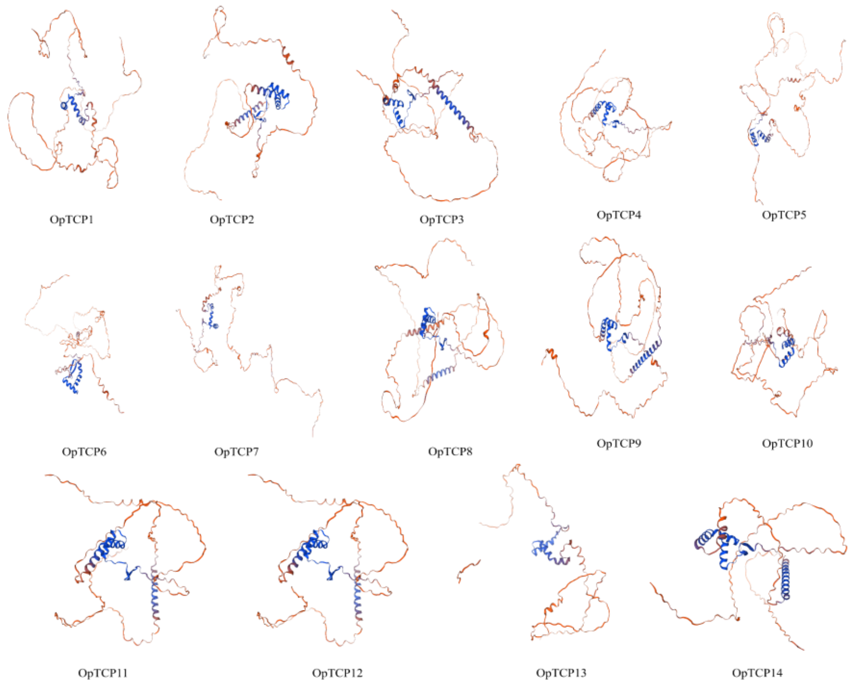

Figure S2: Structural analysis of the tertiary structure of OtTCP genes. Blue indicates consistency greater than 70%, red indicates less than 50%, and yellow indicates consistency between 60%-70%.
